# A Tiny Model for Fast and Precise Ship Detection via Feature Channel Pruning

**DOI:** 10.3390/s22239331

**Published:** 2022-11-30

**Authors:** Yana Yang, Shuai Xiao, Jiachen Yang, Chen Cheng

**Affiliations:** School of Electrical and Information Engineering, Tianjin University, Tianjin 300072, China

**Keywords:** ship detection, SAR, CNN, channel pruning

## Abstract

It is of great significance to accurately detect ships on the ocean. To obtain higher detection performance, many researchers use deep learning to identify ships from images instead of traditional detection methods. Nevertheless, the marine environment is relatively complex, making it quite difficult to determine features of ship targets. In addition, many detection models contain a large amount of parameters, which is not suitable to deploy in devices with limited computing resources. The two problems restrict the application of ship detection. In this paper, firstly, an SAR ship detection dataset is built based on several databases, solving the problem of a small number of ship samples. Then, we integrate the SPP, ASFF, and DIOU-NMS module into original YOLOv3 to improve the ship detection performance. SPP and ASFF help enrich semantic information of ship targets. DIOU-NMS can lower the false alarm. The improved YOLOv3 has 93.37% mAP, 4.11% higher than YOLOv3 on the self-built dataset. Then, we use the MCP method to compress the improved YOLOv3. Under the pruning ratio of 80%, the obtained compressed model has only 6.7 M parameters. Experiments show that MCP outperforms NS and ThiNet. With the size of 26.8 MB, the compact model can run at 15 FPS on an NVIDIA TX2 embedded development board, 4.3 times faster than the baseline model. Our work will contribute to the development and application of ship detection on the sea.

## 1. Introduction

Ship detection has broad application prospects. However, the environment of the sea surface is complex and changeable, which makes it difficult to detect and identify ships on the sea. In the past, researchers used manual feature extraction to classify and locate ships in images. The semantic information is not rich enough, resulting in low accuracy for ship detection. This seriously affects the popularization and application of ship detection [1].

Since AlexNet [2] won the championship in 2012, deep learning has attracted the attention of scholars and been applied in many fields [3,4,5,6,7,8,9], including ship detection. In recent years, most researchers have focused on using convolutional neural networks (CNNs) for ship detection. Cui et al. designed a dense attention pyramid module to obtain rich features. Experiments on the SSDD (SAR Ship Detection Dataset) indicate that the proposed method outperforms other methods for SAR ship detection [10]. Xu et al., exploited a cascaded network including two CNNs for quick and accurate ship detection. The proposed network helps classify and locate ship objects effectively. Experiments show that the method can improve the performance of ship detection [11]. Lin et al., used FCNs (fully convolutional networks) to realize the tradeoff between accurate positioning and feature expression, which contributes to inshore ship detection [12]. Tang et al., designed a hierarchical method for ship detection, including edge enhancement, shape analysis, and features’ studying process. Experiments show that the proposed approach helps reduce the metric of false alarm [13]. Stec B. and Susek W have greatly inspired us in the field of microwave radar and target detection [11,12,13,14,15,16]. Hou et al., designed a framework to separate the ship objects and the background combined with the segmentation and detection task, which helps reduce false detection [17]. Wang et al., integrated CFE architecture, GIOU loss, and data augmentation into the YOLOv3 network for fast and precision ship detection. The whole framework can run at a speed of 29.8 FPS [18]. Wei et al., used faster R-CNN based on dilated convolution to obtain rich feature maps, which can realize precision ship detection on HRSC2016 datasets [19]. Zou et al., utilized the MobileNetV2 structure as the backbone of SSD to obtain the feature maps of ship images, which can effectively reduce the number of parameters of the network and improve the inference speed [20]. Zhang et al., put forward a two-stage ship detection model, by which a novel ship detection method is proposed as well [21]. Yang et al., present graphics processing unit (GPU)-oriented designs for speeding up CFAR detectors, including smallest/greatest-of CFAR and order-statistic CFAR [22].

In general, research on ship detection has made remarkable progress in recent years. However, we have to face two main challenges. Firstly, owing to the complexity of the sea surface environment, it is difficult to obtain rich semantic information of ships, which affects the detection precision. Secondly, the depth of the network used for ship detection is increasing and the number of parameters is skyrocketing, which requires high-performance servers as support and is difficult to run on the embedded platform with limited resources. For the first problem, researchers tend to design efficient feature extraction modules to enrich the semantic feature of ship targets, which will help improve the performance of ship detection. For the second problem, there are many model compression methods proposed that can be used to reduce the number of parameters and model size of detection algorithms; for instance, lightweight network, channel pruning, model quantization, and filter decomposition.

In this paper, we propose a ship detection framework, containing dataset building, detection algorithm optimization, and channel pruning. Our contributions are as follows: (1) We build a SAR ship image database, which provides data support for SAR ship detection. (2) We construct improved YOLOv3 by integrating SPP (spatial pyramid pooling), ASFF (adaptively spatial feature fusion), and DIOU-NMS (distance-IoU non-maximum suppression) module. Experiments on the self-built database show that the improved YOLOv3 obtains 4.11% mAP higher than original YOLOv3. (3) We use the MCP method to automatically search the optimal pruned model. The compressed model has only 6.7 M parameters, running at 15 FPS on NIVDIA TX2. Experiments indicate that channel pruning can reduce the number of parameters and accelerate the detection speed.

## 2. Materials and Methods

### 2.1. Dataset

In this paper, experiments are carried out on SAR ship detection. In order to meet the requirement of training the ship detection model, we build an SAR ship database for experiments.

As for the existing SAR ship datasets, such as the SAR Ship Detection Dataset, Ship_SAR_dataset, SSDD, RadarSAT-2, and Sentinel-1, there are many problems that affect the detection precision. Firstly, the number of images is not big enough, lacking diversity (such as height, distance, and so on). Secondly, some images lack labelling information or the labelling format is not uniform. In addition, the resolution of some images is too large for network training. Thus, we build a SAR ship dataset through data preprocessing, data annotation, and information correction steps to meet the demand of ship detection. Some images are labelled with XML files. Thus, we label those images that do not have annotation.

Ship_SAR_dataset uses high-resolution SAR data and Sentinel-1 SAR data as the main data source. A total of 102 scenes of Gaofen No. 3 and 108 scenes of Sentinel-1 SAR images are used to build a high-resolution SAR ship target depth learning sample library. At present, the in-depth learning sample library contains 43,819 ship slices. The imaging modes of Gaofen-3 are Ultra Fine Stripmap (UFS), Fine Strip Map 1 (FSI), Full Polarization 1 (QPSI), Full Polarization 2 (QPSII), and Fine Strip Map 2 (FSII). The resolutions of these five imaging models are nominal resolution. The imaging modes of Sentinel-1 are strip mode (S3 and S6) and wide imaging mode.

The SSDD dataset contains multiscale SAR ships in different sensors, polarization modes, image resolutions, and scenes. It has 1160 images in total, where the training set includes 928 images and the test set includes the remaining 232 images.

OpenSARShip is a dataset dedicated to Sentinel-1 ship interpretation. The OpenSARShip, providing 11,346 SAR ship chips integrated with automatic identification system messages, has five essential properties: specificity, large scale, diversity, reliability, and public availability. These properties ensure that the OpenSARShip achieves its objectives.

A C-band fine quad-polarization Radarsat-2 dataset acquired over Hong Kong area, with a pixel space of 4.7 m × 4.8 m (range × azimuth), is used in this section. The Radarsat-2 dataset (283 × 458 pixels) contains 457 ship targets. The smallest ship target in these two datasets is about 6 m in width and 26 m in length and occupies 12 pixels in a manually marked ground-truth map.

#### 2.1.1. Data Preprocess

We establish the SAR ship detection dataset based on the SAR Ship Detection Dataset, Ship_SAR_dataset, SSDD, RadarSAT-2, Sentinel-1, and other SAR Ship image datasets. Firstly, images in the above dataset are manually screened. Some unqualified images (such as excessive blur, without targets, and so on) are eliminated. Then, the images with different heights and distances of ship targets (range of 1–4 m) are selected. OpenCV is used to resize and cut out some images that do not meet the requirement of resolution.

**Figure 1:**
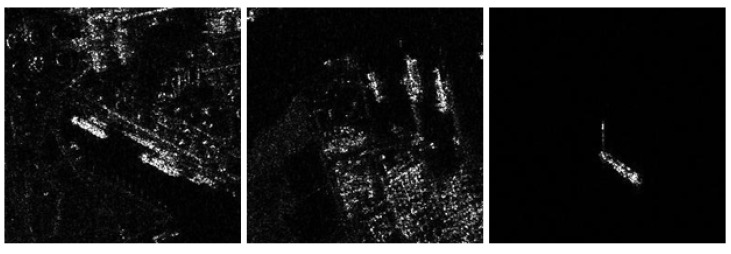
Some examples of the self-built dataset. The scene types include ports, islands and reefs, and sea surfaces with different levels of sea conditions. The targets cover thousands of ships in more than ten categories, such as transport ships, oil tankers, and fishing boats.

After image screening and clipping procedure, we obtain a SAR ship detection dataset including 11,708 SAR images, which contains ships with different heights and distances. After labelling, the self-built dataset can be used for training and testing of SAR ship detection model. Figure 1 shows the examples of our SAR dataset.

#### 2.1.2. Information Correction

In this procedure, we need to manually check whether there is mislabeling, missing labeling, and other situations, and correct the mislabeling. When labeling is complete, we check whether one image corresponds to an XML file. After all the above steps are completed, the SAR ship detection database containing 11,708 images is established, which can be used for subsequent model training and testing.

### 2.2. The Overall Framework

**Figure 2:**
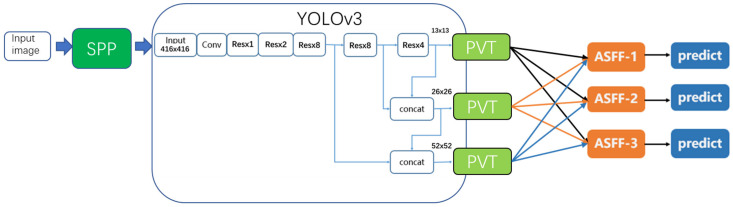
The overall framework of our work. SPP means integrating spatial pyramid pooling, ASFF means adaptive spatial feature fusion and the detection algorithm is based on the YOLOv3 network.

We can use the self-built SAR ship dataset for ship detection and identification. Figure 2 shows the overall framework of this study.

After building the SAR ship detection dataset, we optimize the detection algorithm based on the YOLOv3 [23] network, integrating spatial pyramid pooling (SPP), adaptive spatial feature fusion (ASFF), and DIOU-NMS module. The detection algorithm after optimizing can effectively enrich the feature information of ship targets, which is beneficial to boost the ship detection performance and obtain higher precision. Then, channel pruning is used to reduce the number of parameters and lower the model size of the optimized detection algorithm. The model after pruning can run at a higher detection speed compared with the uncompressed model. Finally, we transplant the compressed model to the NVIDIA TX2 embedded development board.

### 2.3. Improved Ship Detection Algorithm

The fickle sea surface environment makes it difficult to extract ship features from SAR images, leading to the low detection precision. In this work, we add the SPP and ASFF module into the YOLOv3 network to acquire multifarious features of ship targets. In addition, we use DIOU-NMS to filter numerous bounding boxes, which contributes to lowering the false alarm of ship detection.

#### 2.3.1. YOLOv3 Network

**Figure 3:**
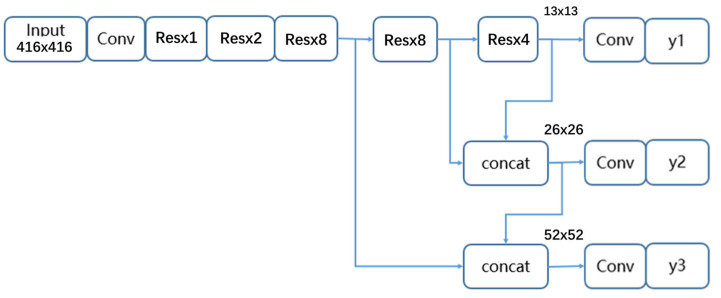
The architecture of YOLOv3. The “Conv” unit represents the common convolutional layer. The “Res” unit represents the shortcut structure borrowed from ResNet. It contains two convolutional layers and one residual layer. “x8” means this unit will repeat eight times. “concat” represents the concatenation layer, which can concatenate two inputs at the channel dimension. Units y1, y2, and y3 represent three detection layers.

YOLOv3, a fully convolutional network, is the third generation of the YOLO [24] series. Figure 3 exhibits the architecture of YOLOv3.

YOLOv3 belongs to a one-stage detection algorithm, which can classify and locate objects in images through a single convolutional network. It uses DarkNet53 as its backbone to extract feature maps of images, which outperforms the ResNet50 [25] and DarkNet19 [26] module. Taking a resolution of 416 × 416 as an example, it can obtain three different scales of feature maps, including 13 × 13, 26 × 26, and 52 × 52. Multi-scale detection is performed according to these feature maps of different sizes. To improve the detection performance of small targets, YOLOv3 borrows the FPN module to fuse different feature maps. In Figure 3, the “Conv” unit represents the common convolutional layer. The “Res” unit represents the shortcut structure borrowed from ResNet. It contains two convolutional layers and one residual layer. “x8” means this unit will repeat eight times. “concat” represents the concatenation layer, which can concatenate two inputs at the channel dimension. Units y1, y2, and y3 represent three detection layers. According to the preset anchor boxes, many bounding boxes are obtained from feature maps. Then, non-maximum suppression (NMS) is used to filter redundant bounding boxes and output the final detection results.

#### 2.3.2. SPP Module

**Figure 4:**
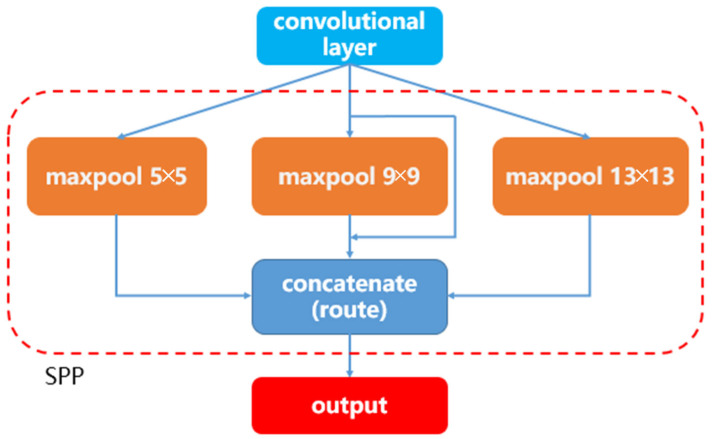
The architecture of the SPP module. The “maxpool” represents the max pooling layer. “concatenate” represents the concatenation layer, which can concatenate two inputs at the channel dimension.

We add three SPP modules in front of the detection layers. The architecture of the SPP module is shown in Figure 4. The SPP module consists of three different formats of the maxpool operation (including 5 × 5, 9 × 9, and 13 × 13). It can fuse the output feature maps of the three maxpool layers. The SPP module has some advantages. Firstly, it can output a fixed size of feature maps, which will solve the problems caused by different sizes of input images. Secondly, the SPP module extracts features from different aspects and concatenates them in the following. This can improve the richness of feature maps and make the algorithm more robust [27]. As SPP can cope with different aspect ratios and sizes of input images, it improves the image’s scale-invariance and reduces over-fitting. The output of SPP is a 416 × 416 image as the input of YOLOv3.

#### 2.3.3. ASFF Module

**Figure 5:**
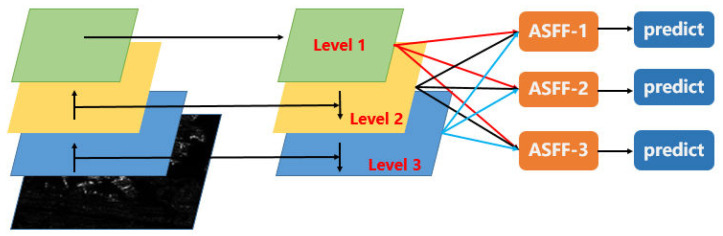
The architecture of the ASFF module. The feature maps of level 1,2,3 are expressed as $X^{1},X^{2},X^{3},\;$respectively, which can be generated using the pyramid vision transformer.

In the original YOLOv3, the concatenation operation is used in the FPN module. This does not take full advantage of the features with different scales. So, we integrate ASFF into YOLOv3 and we replace FPN using a pyramid vision transformer. Figure 5 shows the architecture of the ASFF module.

The transformer is considered as a graph modeling method. The graph is fully connected and the relationship between nodes is learned through the data-driven method. As any concept (whether concrete or abstract) can be represented by nodes in the graph, and the relationship between concepts can be described by edges on the graph, transformer modeling has strong universality. In the task of computer vision, convolution usually deals with the relationship between pixels, that is, local modeling. However, most tasks are designed to objects, that is, the specific structure in the picture, which requires the network to model the relationship between object structure and pixels, or even object to object modeling, and there is no good modeling method. However, because of its universal modeling ability, the attention unit in the transformer can be applied to the modeling of all of these basic relationships.

ASFF can fuse different scales of feature maps [28]. In Figure 5, the feature maps of level 1,2,3 are expressed as $X^{1},X^{2},X^{3},\;$respectively, which can be generated using the pyramid vision transformer. The calculation process of ASFF module can be described by Equation (1):

(1) $$y^{l}=\alpha^{l}*X^{1\to l}+\beta^{l}*X^{2\to l}+\gamma^{l}*X^{3\to l},l=1,2,3$$

In Equation (1), $y^{l}$(l=1,2,3) represents the output of ASFF-*l*. Taking ASFF-1 as an example, $y^{1}$ is obtained by the weighted sum of $X^{1\to 1},X^{2\to 1},X^{3\to 1}$. To be specific, $X^{1\to 1}\;$is the same as $X^{1}$. For $X^{2\to 1}$, $X^{2}$ is twice the size of $X^{1}$, so 3*3 convolution with stride = 2 is used to convert $X^{2}$ to $X^{2\to 1}$. For $X^{3\to 1}$, we should use 3*3 convolution and the maxpool operation to lower the size of $X^{3}$ and obtain $X^{3\to 1}$. For ASFF-3, we need use the interpolation operation on $X^{1},X^{2}\;$to expand the size of feature map and obtain $X^{1\to 3}$, $X^{2\to 3}$. As for the weight parameters α, β, and γ, borrowed from the attention mechanism, we acquire the weight parameters α, β, and γ according to three feature maps $X^{1},X^{2},X^{3}$. We apply 1 × 1 convolution on three feature maps and then use the global max-pooling operation to obtain three attention coefficients. Finally, softmax is used to normalize coefficients and obtain the weight parameters α, β, and γ.

The input of ASFF is the feature maps of the pyramid vision transformer and the output of ASFF is the target prediction of the input image.

#### 2.3.4. DIOU-NMS Module

In the original NMS, the IOU indicator is used to suppress redundant detection boxes. However, only overlapping regions are considered and incorrect suppression occurs often, which may lead to the degradation of detection precision [29]. Unlike the original NMS, DIOU-NMS takes into account not only the value of the IOU, but also the distance between the center points of two boxes, and uses Equation (2) to determine whether a bounding box is deleted:

(2) $$\left\{ \begin{array}{l} s_{i}=\left\{ \begin{array}{l} s_{i},IOU-R(M,B_{i})<\varepsilon \\ 0,IOU-R(M,B_{i})\geq \varepsilon \end{array}\right.\\ R(M,B_{i})=\frac{d^{2}(M,B_{i})}{c^{2}} \end{array}\right.$$

**Figure 6:**
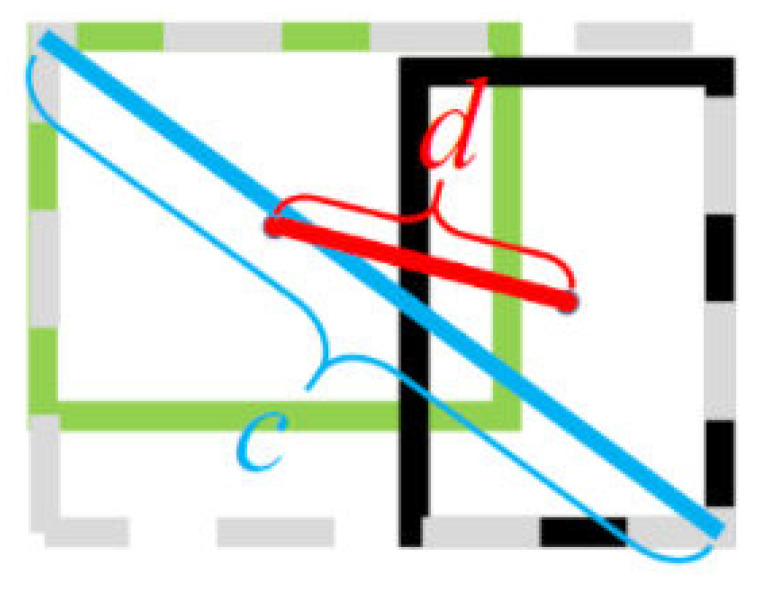
The schematic diagram of calculation process *R.* d is the Euclidean distance and c represents the diagonal length of the smallest box containing two boxes.

In Equation (2), $s_{i}$ is the score value of *i*-th bounding box. IOU,R represent the value of intersection over union and the distance between the center points of two boxes respectively. *M* is the maximum value of score of bounding boxes. The calculation process of distance *R* is shown in the second equation in Equation (2) and Figure 6. d(·) is the Euclidean distance and *c* represents the diagonal length of the smallest box containing two boxes. We use DIOU-NMS to replace the original NMS, in order to achieve a higher detection precision.

The input of DIOU-NMS is a network generated prediction box and the output is the score size of each prediction box. For prediction boxes with similar distances, the maximum value is taken as the prediction output.

### 2.4. Focal Loss

To reduce false detection, we introduce focal loss. Although the OHEM algorithm increases the weight of misclassified samples, it ignores the samples that are easy to classify.

Therefore, for the problem of category imbalance, we introduce focal loss, which is modified on the basis of standard cross entropy loss. This function can reduce the weight of samples that are easy to classify, so that the model can focus more on samples that are difficult to classify during training.

(3) $$FL\left(p_{t}\right)=-\alpha_{t}{\left(1-p_{t}\right)}^{\gamma }log\left(p_{t}\right)$$

### 2.5. Channel Pruning

**Figure 7:**
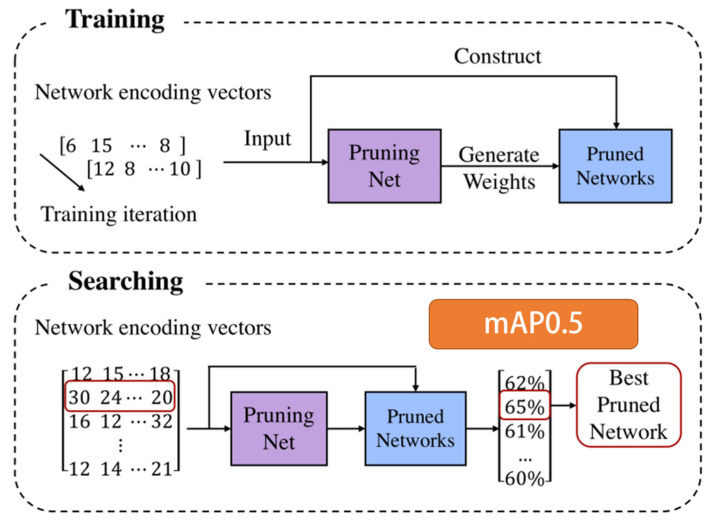
The process of the meta channel pruning method (MCP). At each iteration, a network encoding vector is randomly generated. The Pruned Network is constructed accordingly. The PruningNet takes the network encoding vector as input and generates the weights for the Pruned Network. Searching for the best Pruned Network. We construct many Pruned Networks by varying network encoding vector and evaluate their goodness on the validation data with the weights predicted by the PruningNet. No finetuning or re-training is needed

The input of MCP is a trained network model and the output is a network model with reduced parameters after pruning. Common channel pruning methods require setting some experiment parameters manually [30,31]. In recent years, with the rise of AutoML, it has provided a new solution for pruning [32]. Inspired by meta learning, we construct a model compression method based on it, named meta channel pruning (MCP). Figure 7 exhibits the process of MCP.

The MCP method consists of two steps, training and searching. Firstly, a PruningNet is trained, which can generate weights when giving a pruning strategy to obtain pruned networks. The pruning strategies can be written as ($m_{1},m_{2},\ldots ,m_{l}$), representing the number of channels for the *l*-th layer, respectively. This process of generating weights can be written as Equation (4).

(4) $$W=\mathrm{Pr}uningNet(m_{1},m_{2},\ldots ,m_{l})$$

In Equation (4), *W* means the weights generated by Pr*uningNet*. A block in Pr*uningNet* consists of two FC layers. In the process of back propagation, the gradient of weights in Pr*uningNet* is calculated directly. In this stage, Pr*uningNet* mainly learns to generate weights according to the given pruning strategy. So, in this phase, the weights of Pr*uningNet* will be updated in the process of back propagation. The gradient of weights in pruned networks will not update.

Secondly, we can search pruned networks. After training *PruningNet,* many different pruned networks will be acquired when giving numerous pruning strategies. At the beginning of searching, many strategies are randomly chosen to generate weights through *PruningNet* to obtain the metric mAP on the verification set. The top K strategies with the highest value are selected and new strategies will be acquired using crossover and mutation methods. The optimal pruned network can be obtained by iterating this process repeatedly.

According to the MCP method, we can obtain the best compact detection model automatically compared with the common channel pruning methods, and ease the trouble of manual design.

### 2.6. Embedded Deployment

After channel pruning, we transplant the compressed model to the NVIDIA TX2 embedded development board.

With the advantages of lightweight and convenience, TX2 has been widely used in automatic driving, precision agriculture, and other areas in recent years. It has 256-core Pascal GPU and 8 GB memory. Its performance is comparable to that of a personal computer and we can deploy a variety of artificial intelligence algorithms on TX2, such as image classification, object detection and segmentation, and so on. Owing to limited computing resources, we cannot train the detection model on the NVIDIA TX2 development board. Instead, we should only test the detection model obtained by training process on TX2. When testing on TX2, we should reduce the batch size and directly call the parametered model to detect ships on the testing set at maximum power consumption.

## 3. Results

### 3.1. Experiment Setup

For experiments of improved ship detection algorithm, the detailed running environment includes Python 3.7, CUDA 10.1, CUDNN 7.6.5, PyTorch 1.6, and other Python packages, such as numpy, matplotlib, opencv, and so on. Python was designed by Guido van Rossum of the Netherlands Institute for Mathematical and Computer Science Research in the early 1990s. The number of images in the self-built dataset is 11,708. It is divided into training and testing sets according a 8:2 ratio. Precision and recall are the common metrics in the object detection task. Precision means how many of the detected objects are correct. We can calculate by Precision = TP/(TP + FP). TP means the number of correct detected objects. (TP + FP) means the number of all the detected objects. Recall indicates how many objects are correctly detected. We can calculate Recall = TP/(TP + FN). (TP + FN) means the number of ground truths. In our experiments, the metric mAP0.5 is used to measure detection performance, which is a comprehensive indicator taking into account both precision and recall. We carry out ship detection experiments based on YOLOv3, with an input resolution of 416 × 416.

As for experiments of MCP, the software environment is the same as for ship detection. We set the pruning ratio of 50% and 80% to compare the pruning performance under different conditions. The pruning ratio indicates how many channels will be pruned compared with those channels involved in pruning. Some channels do not need be pruned, such as the convolutional channels next to the detection layers.

### 3.2. Results of Improved Ship Detection

Table 1 shows the results of improved ship detection. SPP, ASFF, and DIOU-NMS modules are integrated into YOLOv3, named improved YOLOv3 in our experiments. We compare the performance with original YOLOv3 and SSD [33]. In the experiments, the input resolution of YOLOv3 and improved YOLOv3 is 416 × 416, while SSD is 320 × 320.

**Figure 8:**
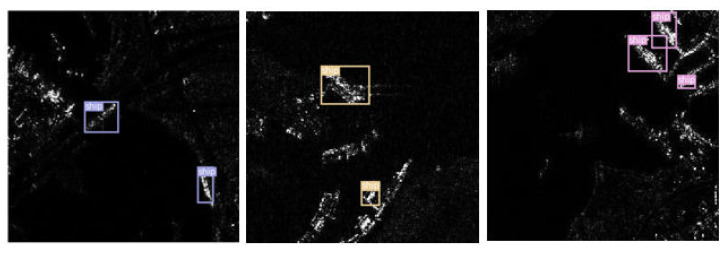
Detection results of improved YOLOv3. Different color means different types of the ships.

From Table 1, for precision, recall [34], and mAP0.5, the improved YOLOv3 is 4. 32%, 4.33%, and 4.19% higher than original YOLOv3, respectively. In addition, the improved YOLOv3 also outperforms SSD algorithm in terms of the three indicators. The experimental results indicate that the improved YOLOv3 can obtain better ship detection performance compared with original YOLOv3 and the SSD network. The SSD network lacks a feature pyramid structure; it directly detects different scales of feature maps. Differently, YOLOv3 contains FPN; it concatenates two roads of feature maps for multi-scale detection. Thus, YOLOv3 outperforms SSD. In addition, we integrated SPP, ASFF, and DIOU-NMS into YOLOv3 to form improved YOLOv3. SPP and ASFF can extract more enriched semantic feature of ships, while DIOU-NMS helps lower the false detection of ship detection. Hence, improved YOLOv3 achieves a higher detection performance than the original YOLOv3 network. Figure 8 shows some detection results of improved YOLOv3.

### 3.3. Results of Channel Pruning

The experiment results of the MCP method are shown in Table 2. The second row means the performance of uncompressed improved YOLOv3. We compare the performance change when the pruning ratio is 50% and 80%. Before pruning, we should determine how many channels will participate in the pruning process. The channels in some layers cannot be pruned. The pruning ration means how many channels will be pruned of those channels participating in the pruning process. The compression ratio indicates a decline in the proportion according to model volume. The pruning ratio indicates the reduction in channels. The compression ratio represents the reduction in model size.

According to Table 2 (“--” means None, it is the base line for others.), when the pruning ratio is 50%, the number of parameters of improved YOLOv3 can degrade from 62 M to 10.4 M, with a 0.34% performance loss. The compression ratio can reach 83.2%.

As for the pruning ratio of 80%, we can obtain the compression ratio of 89.2%, with only 6.7 M parameters. Meanwhile, the detection performance degradez from 93.09% to 92.81%. Finally, we transplant this compressed model with a size of 26.8 MB to the TX2 development board, which can run at a speed of 15 FPS, 4.3 times faster than the uncompressed model. The experimental results show that the MCP method can effectively reduce the number of parameters and model size. Channel pruning also helps accelerate the detection speed.

In order to verify that the advancement of our proposed method does not come from dataset bias, we also conducted a set of comparative experiments on public datasets, and the results are shown in Table 3. The results show that our method also has high performance on public datasets.

### 3.4. Ablation Experiments

Our proposed model is divided into multiple modules, the SPP module, ASFF module, and DIOU-NMS module. In this section, we will design experiments to verify whether these three modules have certain effects. Among them, the model in the experiment with or without the SPP module constitutes a set of controls, ASFF and FPN constitute a set of controls, and DIOU-NMS and NMS constitute a set of controls. The experimental results are shown in Table 4, which shows that SPP, ASFF, and DIOU-NMS all improve the model performance to a certain extent, and this gain is additive.

There are two major challenges to ship detection. Firstly, it is difficult to extract the features of ship targets, leading to the low detection performance. Secondly, detection models usually have numerous parameters, which is unaffordable for some equipment with limited resources [35]. Therefore, our work in this paper contributes to solving two problems. To improve the ship detection precision, we integrate the SPP, ASFF, and DIOU-NMS module into original YOLOv3. In concrete, using SPP and ASFF helps obtain rich semantic information of ship targets. Features can be fully taken advantage of by fusing features of different scales. With original NMS replaced by DIOU-NMS, false alarms decrease. The experimental results obviously show that improved YOLOv3 outperforms the YOLOv3 network.

To achieve a compact model, the MCP method is used for model compression. To verify the effectiveness of the method, we compare MCP with two other channel pruning methods, NS [36] and ThiNet [37]. Table 5 shows the results of the comparison. Comparative experiments are carried out under the pruning ratio of 80%.

The model after the NS method has 8.4 M parameters, with mAP0.5 of 91.89%. When using ThiNet method, the compressed model has 7.5 M parameters. We can find that MCP can obtain a model with fewer parameters and higher detection performance than NS and ThiNet. In the end, we deploy the compressed model after MCP to the NVIDIA TX2 embedded development board, running at 15 FPS with an input resolution of 416 × 416. It can run 4.3 times faster than the baseline model.

In the future, we want to design efficient module based on the transformer to make full use of different scales of feature maps [38], and then use the genetic evolutionary algorithm to search the optimal compressed model automatically in one step [39].

## 4. Conclusions

Facing the problems of extracting rich features and containing huge amounts of parameters for a ship detection model, in this work, we design a framework including improved ship detection and channel pruning. In concrete, we build a SAR ship detection database on our own. Then, we integrate SPP and ASFF based on YOLOv3 to increase the diversity of feature maps. DIOU-NMS is used to decrease false alarms. The improved YOLOv3 has 93.37% mAP, 4.11% higher than that of original YOLOv3. Afterwards, we construct the MCP method to compress the improved YOLOv3. The compressed model has a compression ratio of 89.2%, with a 0.62% mAP decline. Finally, the compact model can run 15 FPS on NVIDIA TX2, 4.3 times faster than the uncompressed model.

## Figures and Tables

**Table 1:** Results for improved ship detection.

	Input Resolution	Precision	Recall	mAP0.5
YOLOv3	416	89.45%	89.95%	89.26%
SSD	320	87.89%	88.02%	87.05%
YOLO + SPP + ASFF + DIOU (Ours)	416	93.77%	94.28%	93.45%

**Table 2:** Results for the MCP method.

	Parameters	Model Size	mAP0.5	Compression Ratio
YOLO + SPP + ASFF + DIOU (baseline)	62 M	248 MB	93.45%	--
Pruning—50%	10.4 M	41.6 MB	93.09%	83.2%
Pruning—80%	6.7 M	26.8 MB	92.81%	89.2%

**Table 3:** Results for other datasets.

Method	HRSC2016		SSDD+		AIR-SARShip-1.0	
	AP50	mAP	AP50	mAP	AP50	mAP
RR-CNN	75.7	46.1	90.2	63.6	84.2	52.1
RBox-CNN	76.0	46.9	90.4	64.2	85.3	52.9
ReDet	76.2	47.1	90.5	64.4	85.9	53.6
RBFA-Net	76.5	47.4	90.8	64.8	87.7	53.9
YOLO + SPP + ASFF + DIOU (Ours)	**77.6**	**48.2**	**91.1**	**65.1**	**88.2**	**54.4**

**Table 4:** Results of ablation experiments.

SPP	ASFF	FPN	Focal Loss	Precision	Recall	mAP0.5
×	×	×	×	89.45%	89.95%	89.26%
√	×	×	×	90.17%	90.24%	89.84%
√	√	×	×	91.96%	91.84%	90.89%
√	√	√	×	93.49%	94.02%	93.37%
√	√	√	√	93.77%	94.28%	93.45%

**Table 5:** Comparison of the channel pruning methods.

	Parameters	Model Size	mAP0.5	Compression Ratio
YOLO + SPP + ASFF + DIOU (baseline)	62 M	248 MB	93.37%	--
NS-80%	8.4 M	33.6 MB	91.89%	86.5%
ThiNet-80%	7.5 M	30 MB	92.16%	88%
MCP-80%	6.7 M	26.8 MB	92.75%	89.2%

## Data Availability

Data available on request from the authors.
